# The crystal structure of the Hazara virus nucleocapsid protein

**DOI:** 10.1186/s12900-015-0051-3

**Published:** 2015-12-29

**Authors:** Rebecca Surtees, Antonio Ariza, Emma K. Punch, Chi H. Trinh, Stuart D. Dowall, Roger Hewson, Julian A. Hiscox, John N. Barr, Thomas A. Edwards

**Affiliations:** Public Health England, Porton Down, Salisbury, Wiltshire SP4 0JG UK; School of Molecular and Cellular Biology, Faculty of Biological Sciences, and Astbury Centre for Molecular and Structural Biology, University of Leeds, Leeds, LS2 9JT UK; Present address: Sir William Dunn School of Pathology, University of Oxford, South Parks Road, Oxford, OX1 3RE UK; Present address: Department of Infection Biology, Institute of Infection and Global Health, University of Liverpool, Liverpool, UK

**Keywords:** Hazara, CCHFV, Nairovirus, Nucleocapsid protein, RNP

## Abstract

**Background:**

Hazara virus (HAZV) is a member of the *Bunyaviridae* family of segmented negative stranded RNA viruses, and shares the same serogroup as Crimean-Congo haemorrhagic fever virus (CCHFV). CCHFV is responsible for fatal human disease with a mortality rate approaching 30 %, which has an increased recent incidence within southern Europe. There are no preventative or therapeutic treatments for CCHFV-mediated disease, and thus CCHFV is classified as a hazard group 4 pathogen. In contrast HAZV is not associated with serious human disease, although infection of interferon receptor knockout mice with either CCHFV or HAZV results in similar disease progression. To characterise further similarities between HAZV and CCHFV, and support the use of HAZV as a model for CCHFV infection, we investigated the structure of the HAZV nucleocapsid protein (N) and compared it to CCHFV N. N performs an essential role in the viral life cycle by encapsidating the viral RNA genome, and thus, N represents a potential therapeutic target.

**Results:**

We present the purification, crystallisation and crystal structure of HAZV N at 2.7 Å resolution. HAZV N was expressed as an N-terminal glutathione S-transferase (GST) fusion protein then purified using glutathione affinity chromatography followed by ion-exchange chromatography. HAZV N crystallised in the P2_1_2_1_2_1_ space group with unit cell parameters *a* = 64.99, *b* = 76.10, and *c* = 449.28 Å. HAZV N consists of a globular domain formed mostly of alpha helices derived from both the N- and C-termini, and an arm domain comprising two long alpha helices. HAZV N has a similar overall structure to CCHFV N, with their globular domains superposing with an RMSD = 0.70 Å, over 368 alpha carbons that share 59 % sequence identity. Four HAZV N monomers crystallised in the asymmetric unit, and their head-to-tail assembly reveals a potential interaction site between monomers.

**Conclusions:**

The crystal structure of HAZV N reveals a close similarity to CCHFV N, supporting the use of HAZV as a model for CCHFV. Structural similarity between the N proteins should facilitate study of the CCHFV and HAZV replication cycles without the necessity of working under containment level 4 (CL-4) conditions.

## Background

The *Bunyaviridae* family of segmented negative stranded (SNS) RNA viruses constitutes a diverse group of over 350 members separated in five genera namely *Hantavirus, Nairovirus*, *Orthobunyavirus*, *Phlebovirus*, and *Tospovirus*. Together these viruses infect a bewildering array of animals and plants, as well as causing serious disease in humans. One of these human pathogens is Crimean-Congo haemorrhagic fever virus (CCHFV), which is a member of the *Nairovirus* genus and is the causative agent of Crimean-Congo haemorrhagic fever (CCHF), a human disease that can progress to haemorrhagic manifestations and death in up to 30 % of cases [1, 2]. CCHFV reservoirs are maintained in a wide variety of both wild and domestic mammals, and the virus is transmitted to humans by either CCHFV-infected ticks of the *Hyalomma* species, or from direct contact with the blood or tissue of an infected human or animal [3–5]. CCHFV is the second most widespread medically important arbovirus after Dengue virus, and is currently endemic or potentially endemic in 52 countries throughout Africa, Asia, the middle east, the Balkans and Europe [6]. A recent *in silico* study predicted the continuing spread of CCHFV to northern European countries (including the UK) based on predicted increases in climate temperature that would lead to an expansion in the habitat suitable for the tick vector [7]. Due to both the extreme pathogenicity of CCHFV in humans, and a current lack of effective preventative or therapeutic measures, CCHFV is classified within Hazard Group 4, requiring the highest level of biological containment.

HAZV is classified in the same serogroup as CCHFV; however, HAZV has not been documented to cause serious disease in humans and consequently is categorised as a hazard group 2 pathogen. The global distribution of HAZV has not been thoroughly investigated, however antibodies against HAZV have been detected in wild rodent sera [8], and HAZV has been isolated from *Ixodes redikorzevi* ticks in Western Pakistan [9]. Experimental infection of several different mammalian species (including various species of mice and rats, guinea pigs, rabbits and donkeys) with both HAZV and CCHFV has resulted in successful virus replication [10]. In both cases the only animals that display clinical symptoms with fatal outcome are suckling mice and interferon receptor knockout mice [11, 12]. Given the similarity in CCHFV and HAZV disease progression in the interferon receptor knockout mouse model, it is thought that HAZV could represent a valid model for CCHFV infection, enabling the investigation of this serogroup of viruses and the development of antivirals without having to work in a containment level 4 (CL-4) environment.

The genomes of CCHFV and HAZV comprise three negative sense RNA segments, named small (S), medium (M) and large (L), which encode the nucleocapsid protein (N), the viral glycoproteins (Gn and Gc) and the viral RNA dependent RNA polymerase (L), respectively. As with all SNS RNA viruses, these RNA segments are encapsidated by the nucleocapsid protein (N) to form ribonucleoprotein (RNP) complexes [13]. Genome encapsidation by N is required for multiple stages of the virus life cycle, including replication and transcription of the viral genome, as well as segment packaging during virus assembly [14]. For a comprehensive review on the molecular biology of these viruses refer to [15, 16].

**Fig. 1:**
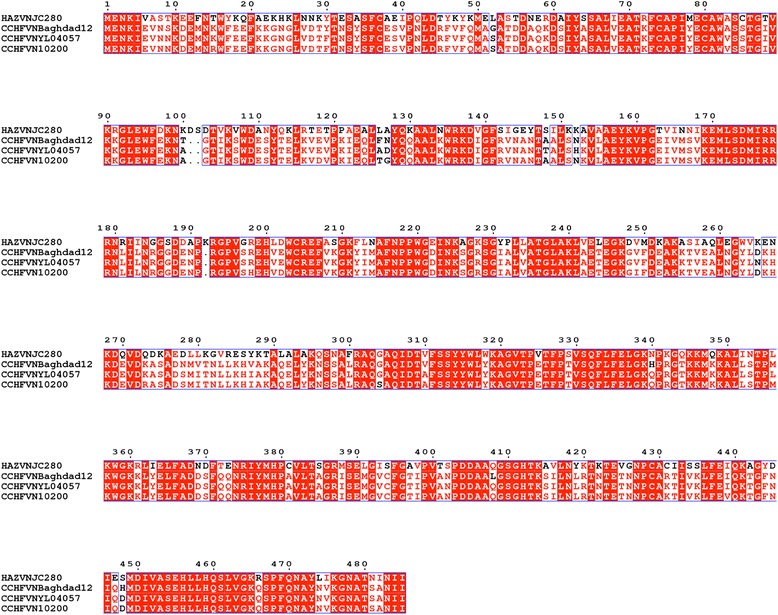
Amino acid sequence alignment of CCHFV N strains Baghdad-12, YL04057 and IbAr10200 with HAZV N strain JC280. Alignment was carried out using Clustal Omega and Espript [38]. Completely conserved residues are white with a red background. Residues which do not share similar properties and are not conserved are black

The crystal structures of the full length CCHFV N protein (residues 1–482) have been reported from several different virus strains. N from the YL04057 strain was determined to a resolution of 2.3 Å [17], N from the Baghdad-12 strain to a resolution of 2.1 Å [18], and N from the IbAr10200 strain to 3.1 Å [19]. These structures revealed two domains; a globular domain and an arm domain, which are linked by a flexible loop. The globular domain of the Baghdad-12 strain is formed from 23 alpha helices which are derived from both the N- and the C-terminal regions of the N protein. Alpha helices from the N-terminus surround the C-terminal alpha helices, which form the core of the globular domain [18]. Two long and three short alpha helices form the arm domain, which extends away from the globular domain and is located in a different position in each of the three CCHFV N crystal forms [18]. In all cases the structure of monomeric, RNA free CCHFV N was determined. CCHFV N and HAZV N share 59 % amino acid sequence identity (Fig. 1), with HAZV N encoding three extra amino acids in comparison to CCHFV N. It is thought that CCHFV N and HAZV N perform the same function in their virus replication cycles, therefore to determine the extent of CCHFV N and HAZV N structural similarities we have solved the crystal structure of full length HAZV N, at a resolution of 2.7 Å.

## Results and discussion

### Expression and purification of HAZV N

HAZV N was expressed as an N terminal glutathione S-transferase (GST) fusion protein (GST-HAZV N) and purified by glutathione affinity chromatography. Following overnight incubation with human rhinovirus (HRV) 3C protease to remove the GST tag, the oligomeric status of HAZV N was investigated using size exclusion chromatography (data not shown). HAZV N eluted as several different species; the protein that eluted most rapidly likely represents HAZV N multimer forms, and the slower eluting and most-abundant protein species likely represents the HAZV N monomer (predicted molecular weight 54 kDa). The cleaved GST co-eluted with the HAZV N monomer due to the fact that GST has a molecular weight of 26 kDa, and dimerises in solution (predicted molecular weight 52 kDa) and so we concluded that SEC was unsuitable for further HAZV N purification.

**Fig. 2:**
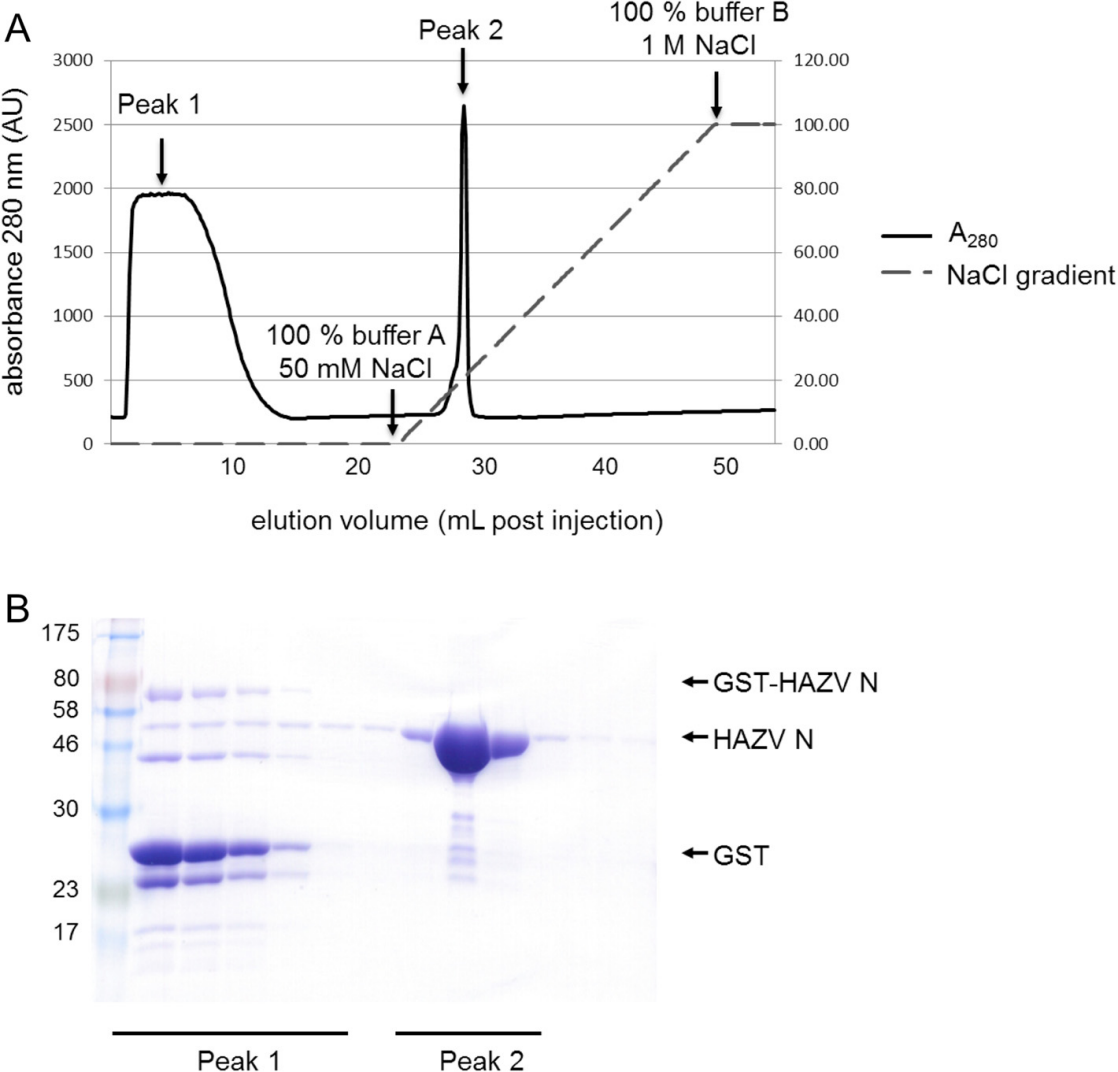
HAZV N purification by ion exchange chromatography. HAZV N was separated from GST-HAZV N and GST using ion exchange chromatography. **a** Protein elution was monitered with an A_280_ absorbance trace; protein that flowed through the column without binding is represented by Peak 1, whereas protein that bound to the column and was then eluted by an increasing NaCl gradient (dashed grey line) forms Peak 2. **b** SDS PAGE analysis of Peak 1 and Peak 2. Peak 2 contains primarily HAZV N, whereas Peak 1 contains a mixture of proteins that flowed through the column without binding

**Fig. 3:**
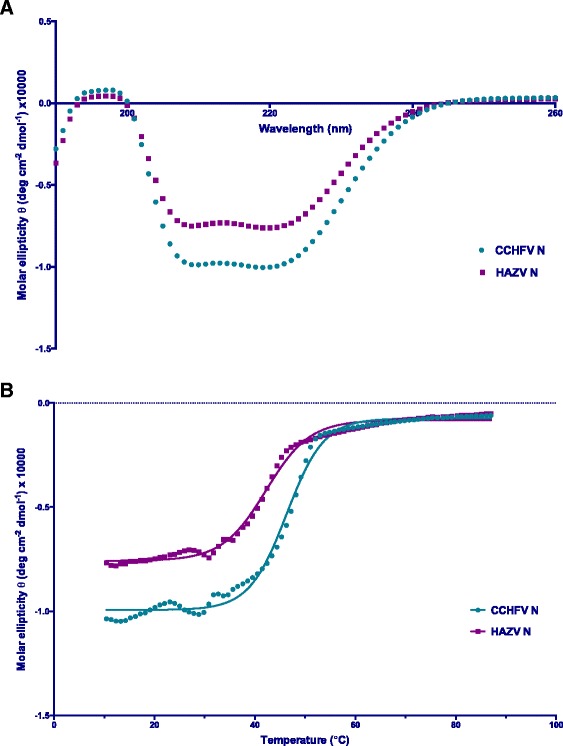
Circular dichroism analysis of CCHFV N and HAZV N. Circular Dichroism was used to analyse the secondary structure of both HAZV and CCHFV N proteins. **a** Ellipticity across the far UV spectra illustrate that both proteins are primarily α – helical, demonstrated by the characteristic minima at 208 nm and 222 nm. **b** The thermal stability of CCHFV and HAZV N proteins were compared by monitoring ellipticity at 222 nm, we find that the melting temperature (Tm) of HAZV N is 42.21 °C and of CCHFV N is 46.30 °C

HAZV N was therefore purified from uncleaved GST-HAZV N and GST by cation exchange chromatography using a Resource S column (Fig. 2). HAZV N has an isoelectric point of 8.73, therefore ion exchange was performed with all buffers equilibrated to pH 7, where HAZV N should be positively charged and bind to the negatively charged Resource S column, and all other proteins should pass through the column without binding (Fig. 2, peak 1). HAZV N was eluted from the column (Fig. 2, peak 2) as the concentration of NaCl was increased from 50 mM to 1 M over 25 column volumes. Cation exchange chromatography removed the majority of protein contaminants leaving only HAZV N, then circular dichroism was used to analyse the thermal stabilities of both HAZV N, and CCHFV N proteins (Fig. 3) as previously described [20]. When monitoring the ellipticity at 222 nm the melting temperature (T_m_) of HAZV N was found to be 42.2 °C and of CCHFV N is 46.3 °C. This indicates both proteins are correctly folded and potentially share a similar secondary structure.

### Crystallisation of HAZV N and structure solution

HAZV N was concentrated to 10 mg ml^−1^ and used in crystallisation trials. Commercially available sparse matrix screens were initially used to determine conditions under which HAZV N crystallised. Successive rounds of optimisation were then performed to determine the condition under which the best diffracting crystals grew; the final diffraction data were collected from crystals that grew in 15 % PEG 10,000, 0.1 M sodium citrate pH 5, 0.1 M NaCl and 2 % dioxane.

After data collection, the structure of HAZV N was solved by the molecular replacement method with the globular domain of CCHFV N (PDB accession number: 4AKL); residues 1–182, and 296–482 as the initial search model. The final structure of the full length HAZV N model was refined to R_cryst_ = 24.2 % and R_free_ = 28.1 % with good geometry: overall MolProbity score = 1.35 (100th percentile for resolution).

**Fig. 4:**
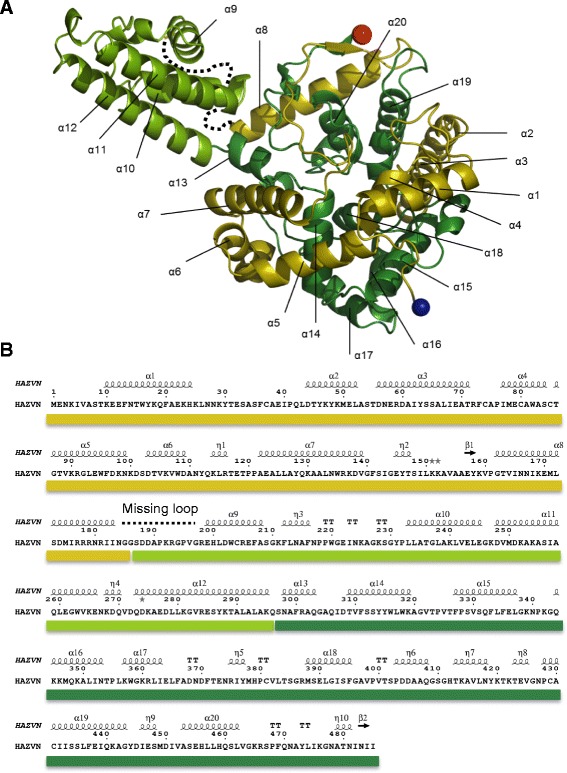
The crystal structure of the HAZV N monomer and alignment of the sequence of HAZV N with the secondary structural elements. **a** Monomer A from the HAZV N AU. A blue sphere indicates the N-terminus and a red sphere indicates the C-terminus of HAZV N. The globular domain contains amino acids residues from the N-terminus (residues 1–186, yellow), and the C-terminus (residues 298–485, dark green). The arm domain (residues 197–297, light green) is linked to C-terminus of the globular domain by a single alpha helix (helix α13), and to the N-terminus by a disordered loop (residues 187–196), which is not visible in this model. Helices are numbered according to (**b**). **b** Alignment of the sequence of HAZV N with the secondary structural elements. Alpha helices are numbered 1–20 from the N-terminus to the C-terminus. Horizontal bars below the sequence are coloured according to (**a**) and indicate the position of the secondary structural elements in (**a**). Helix α1 - helix α8 comprise the N-terminus of the globular domain, helix α9 – helix α12 the arm domain, and helix α13 – helix α20 the C-terminus of the globular domain. (Generated using Espript [38]). _TT_ denotes a strict β-turn and ɳ denotes a helix with 3 residues per turn. All models were generated using Pymol

The crystallographic asymmetric unit (AU) contains four HAZV N monomers. A single HAZV N monomer (monomer A) is presented in Fig. 4. In each of the four HAZV N monomers in the AU, only 475 amino acid residues were built into the model; residues 187–196 are missing in the electron density and are thought to form a disordered loop that links the arm domain to the N terminus of the globular domain. In agreement with the circular dichroism data, HAZV N is mostly alpha helical (helices are numbered according to Fig. 4b) and is composed of two distinct domains, a globular domain and an arm domain. The globular domain is formed from a central core comprising C-terminal helices α13 – α20 (residues 298–485 – dark green in Fig. 4a) surrounded by N-terminal helices α1 - α8 (residues 1–186 - yellow in Fig. 4a). The arm domain (light green in Fig. 4a) extends away from the globular domain, and is formed from two long alpha helices (helix α11 and helix α12), which are supported by a small three-helix bundle (helices α9, α10, and ɳ3). There is a loop on the apex of the arm domain which exposes a potential caspase cleavage site (DQVD), however it is unknown whether HAZV N is cleaved during the course of the virus replication cycle. A single alpha helix (helix α13) provides the only link visible in this model between the arm domain and the globular domain, as the loop linking the N-terminus of the globular domain to the arm domain (residues 187–196) is not visible in the electron density and is presumed disordered.

### Monomer interactions within the HAZV N AU

**Fig. 5:**
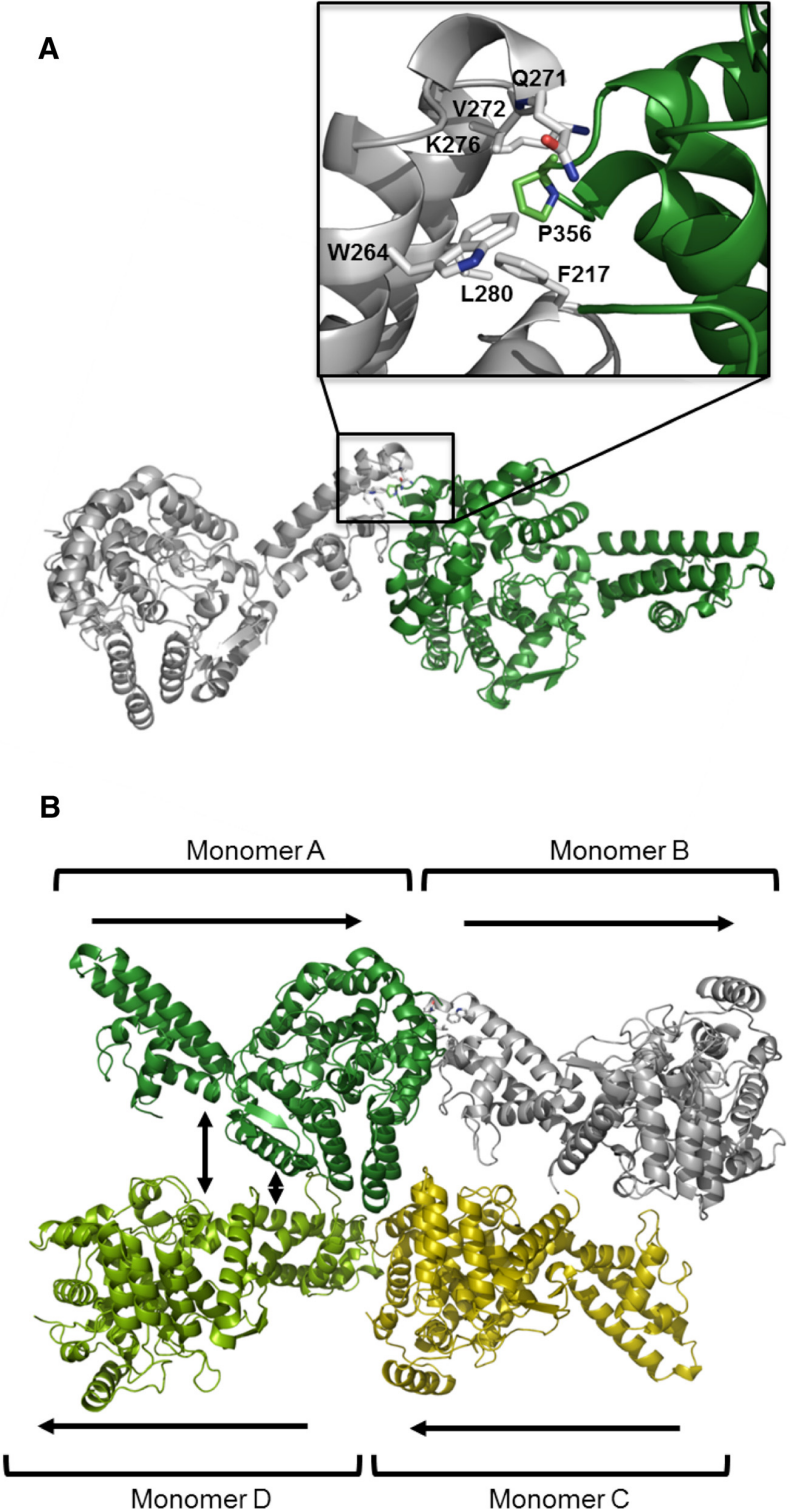
Crystallographic interfaces between adjacent HAZV N monomers in the AU. **a** Interaction between HAZV N monomers is thought to occur between Pro356 on the base of the globular domain, which is buried in a hydrophobic pocket is formed by six residues (Leu280, Trp264, Lys276, Val272, Glu271, and Phe217) of the arm domain of an adjacent monomer. **b** the arrangement of HAZV N monomers in the AU: two pairs of monomers are packed anti-parallel to each other. Electron density for the 4th monomer (light green) was poor compared to the other monomers in the AU. Potential lateral interactions between HAZV N monomers are indicated by vertical arrows. All models were generated using Pymol

The four HAZV N monomers in the AU are arranged as two pairs of monomers running anti-parallel to each other. Within each pair, contact is between six residues from both the apex of the arm domain and the supporting three helix bundle of one monomer (residues Leu280, Trp264, Lys276, Val272, Glu271, and Phe217) and the base of the globular domain of the second monomer (residue Pro356). It is thought residue Pro356 from the base of the globular domain from one monomer is buried within a hydrophobic pocket formed from the six residues on the arm domain of the second monomer (Fig. 5).

Interestingly, HAZV N and the CCHFV N monomers in the AU of all three CCHFV N structures are packed in a similar way, with the base of the globular domain of one monomer making contact with residues on the arm domain of the adjacent monomer. This interaction between adjacent monomers results in the formation of long helical chains of HAZV N and CCHFV N, which are visible throughout each of the four different crystal forms. The helical chains of N have been well described by Wang et al. [19]. The buried surface area at this site is also conserved (HAZV N 1042 Å^2^; PDB 4AKL 1052 Å^2^; 4AQG 977 Å^2^; 3U3I 916 Å^2^). The conserved nature of this interaction between multiple crystal forms suggests it may be a common feature of nairovirus N proteins, and the structures observed in the crystals may reflect those that form during RNP assembly in infected cells. The fact that the pairs of monomers in the HAZV N AU are arranged anti-parallel to each other suggests this potential multimerisation arrangement resembles that recently reported for influenza A virus (IAV) RNPs, which comprise two anti-parallel N-RNA strands connected by a short loop at one end, and associated with the polymerase complex at the other end [21]. However, in contrast to the interaction between the base of one globular domain and the arm domain of the adjacent monomer, the lateral interaction between the pairs of monomers in the HAZV N AU is not conserved and varies between HAZV N and the three CCHFV N crystal forms (Fig. 5b).

### HAZV N protein electrostatic surface potential in relation to proposed function

**Fig. 6:**
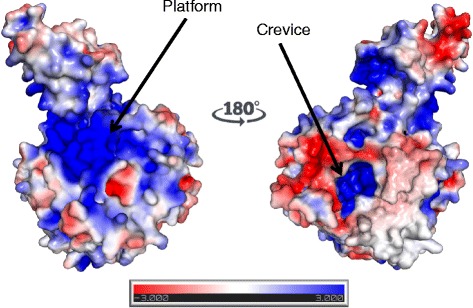
Surface representation of Apo-HAZV N electrostatic potential. The electrostatic surface potential of HAZV N is shown colour coded from red (negative) to blue (positive) in dimensionless units of k_b_.T/e_c_, where k_b_ is Boltzmann’s constant, T is the temperature, and e_c_ is the charge of an electron (generated using the APBS Pymol plug-in [39]). Potential RNA binding sites, the Platform and the Crevice are labeled

As HAZV N is a proposed RNA binding protein, the electrostatic surface potential of HAZV N was analysed to identify potential RNA binding sites. Surface representations of the HAZV N protein colour-coded red to blue according to charge (negative to positive, respectively) are presented in Fig. 6. The surface representation of HAZV N reveals several areas of positive charge (blue), which are often associated with an increase in RNA binding potential. HAZV N has a positively charged ‘platform’ adjacent to the arm domain (Fig. 6, left) in a similar position to a positively charged platform on the surface of the CCHFV N protein. This platform is one of the largest areas of continuous positive charge on the surface of both HAZV N and CCHFV N, and was therefore suggested to be a possible RNA binding site. Another possible RNA binding site is a crevice on the opposite face of the HAZV N monomer to the positively charged platform (rotate HAZV N monomer 180°, Fig. 6, right). This crevice contains positively charged residues and extends across the length of the globular domain from helix α13 at the beginning of the arm domain, to the base of the globular domain. This crevice is also present in the CCHFV N model, and RNA sequestered here could be protected from degradation.

**Fig. 7:**
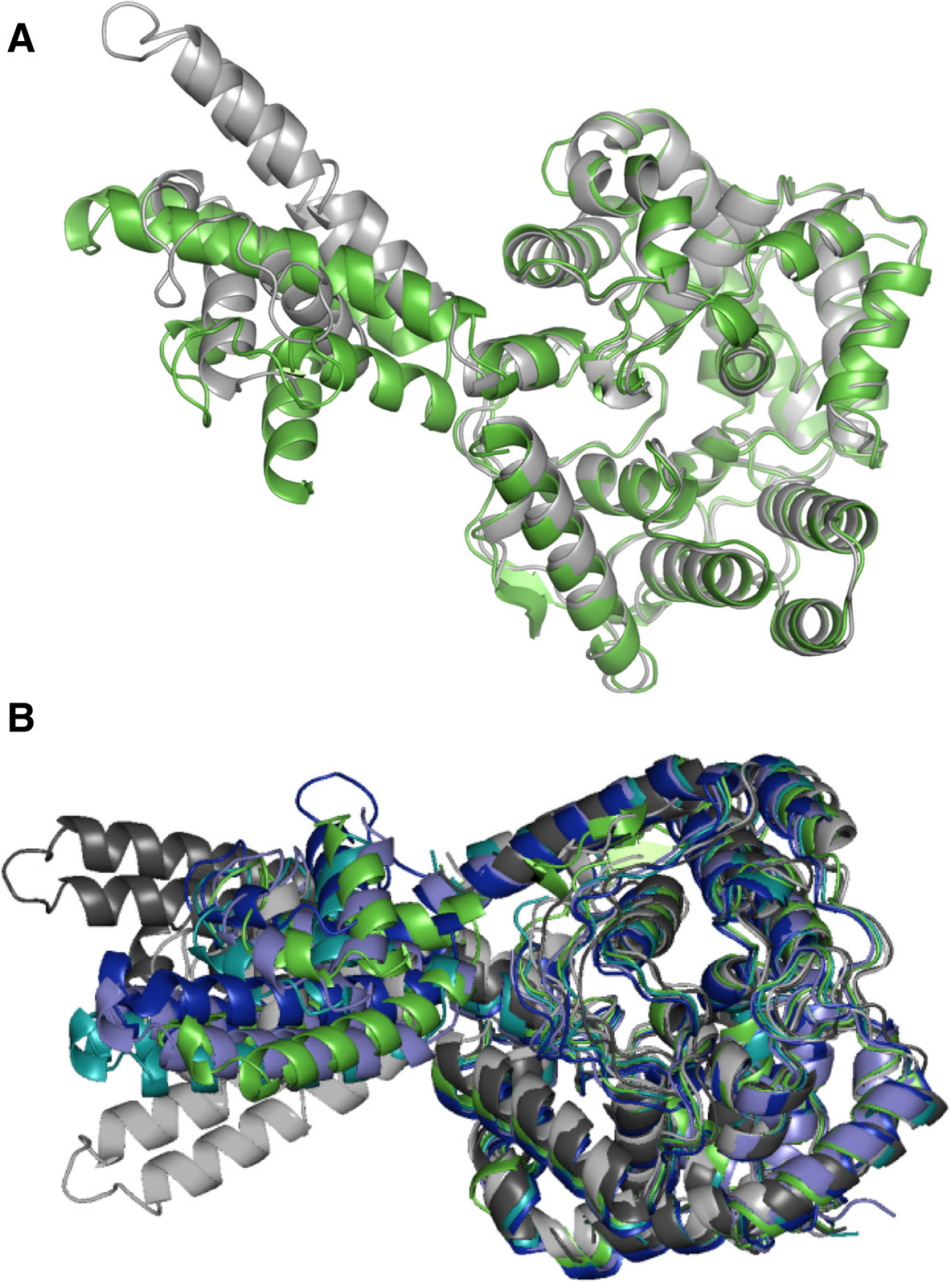
Superposition of CCHFV N and HAZV N. **a** The globular domain of HAZV N (green) and CCHFV N strain Baghdad-12 (grey) superpose very closely. The relative position of the arm domain of CCHFV N is shifted compared to HAZV N via a rotation of 74.0 ° around a pivot at serine 297. **b** Superposition of CCHFV N monomers from different strains YL04057 (dark grey), Baghdad-12 (grey), and IbAr10200 (blue, teal and light blue) with HAZV N (green). All models were generated using Pymol

**Fig. 8:**
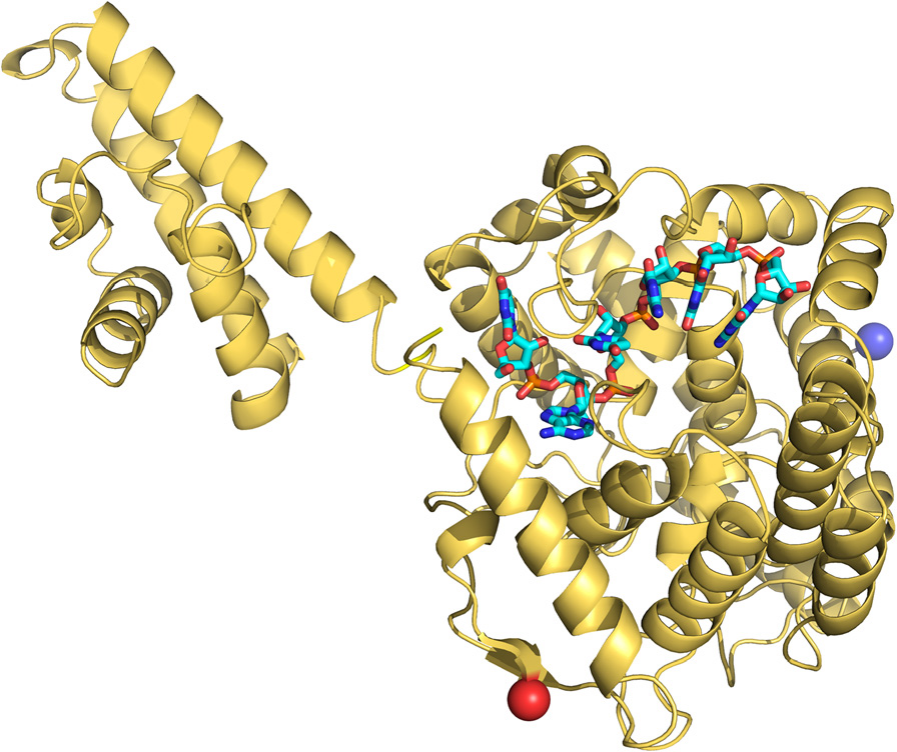
Model of HAZV N bound to RNA. After superposition of the HAZV N core domain with the N structure from Lassa bound to RNA, The Lassa protein has been removed to leave a model for how HAZV N may bind to RNA (generated in PyMol)

We previously revealed structural similarity between the globular domain of the CCHFV N protein and the N-terminal RNA binding domain of the nucleocapsid protein (NP) of Lassa virus (LASV), which is a segmented negative stranded RNA virus classified within the *Arenaviridae* family [18]. Not surprisingly, we show here that the HAZV N protein also is structurally similar to LASV NP. Overlay of HAZV N with RNA-bound LASV NP strongly suggests that the HAZV N protein residues within the ‘crevice’ mediate RNA binding (Fig. 8).

### The structure of HAZV and CCHFV N in relation to other bunyavirus N proteins

HAZV N and CCHFV N likely perform the same function in their viral replication cycles, and share approximately 59 % sequence identity; this is reflected in their structural similarity. Superposition of HAZV N with CCHFV N strain Baghdad-12 (Fig. 7a) reveals that the globular domains of these proteins align extremely closely (RMSD = 0.70 Å, over 368 residues that share 63 % sequence identity), as do the arm domains (RMSD = 0.89 Å over 107 residues that share 48 % sequence identity). However the relative position of the arm domain with respect to the globular domain varies between the two structures (Fig. 7a). The arm domain of CCHFV N is rotated by 74.0° around a pivot at serine 297 when compared with the arm domain of HAZV N resulting in a translation of 27.1 Å of the tip of the arm domain (the Cα of Asp 266).

Indeed, the arm domain adopts a different position in all three CCHFV N crystal structures as well as in the HAZV N structure (Fig. 7b). Different amino acid sequences could account for the change in the position of the arm domain, or the difference in the position of the arm could be related to differences in crystal packing. However the flexibility in the N protein of CCHFV and HAZV that allows the arm domain to adopt different positions is likely a fundamental property of these proteins that allows them to carry out their function effectively.

The structure of the N protein of several members of the *Bunyaviridae* family of segmented RNA viruses have recently been solved, including that of Rift Valley fever virus, Bunyamwera virus, Schmallenberg virus, Leanyer virus, Toscana virus and La Crosse virus [22–27]. Although the nucleocapsid proteins of bunyaviruses are structurally diverse (they do not share common folds) they do all possess at least two separate domains; a largely alpha-helical globular domain with a positively charged groove that binds viral RNA, and another domain that is often arm-like and which is involved in oligomerisation of neighbouring globular RNA binding domains. These domains are linked by highly flexible hinge regions that allow N proteins to adopt a number of different conformations, which enables the formation of RNPs having variable morphologies [28, 29]. It has been proposed that the flexibility of the SNS RNPs may be required for different stages of the virus replication cycle, such as genome replication and segment packaging. For example, the viral genomic RNA strands form open circular pan-handle structures mediated in part by sequence-specific interactions with the viral polymerase [30], as well as complimentary base pairing between the RNA strand termini. When encapsidated by N, it is thought the inherent flexibility of the RNP chain may be essential to allow the physically distant RNP ends to find each other to permit this intra-terminal interaction to occur [28].

## Conclusion

We have determined the crystal structure of HAZV N to a resolution of 2.7 Å and found the structure of HAZV N to be very similar to the structure of CCHFV N, supporting the use of HAZV as a model for CCHFV infection. The N proteins of CCHFV and HAZV both contain a globular domain and an extended arm domain that are linked by a flexible hinge region. Although both domains of CCHFV N and HAZV N superpose on one another closely, the arm is in a different position in each structure. However the arm is also in a different position in all three CCHFV N models, possibly reflecting the inherent flexibility between these two domains. The different arm positions in each model means that the angle between the core of one monomer and the core of the adjacent monomer are different in the helical arrays mediated by the well conserved arm:core interactions (Fig. 5). The different arm position may well reflect a real function, and the differences in helical arrangements of the protomers may reflect different N conformations that form during the viral replication cycle – RNP assembly, transcription and replication for instance. The identification of areas of structural similarity between CCHFV and HAZV N could potentially be exploited in the structure-guided design of small molecule inhibitors, whose efficacy against HAZV could be rapidly tested under CL-2 conditions, prior to testing against CCHFV.

## Methods

### Cloning, expression and purification

The cDNA encoding full length HAZV N was originally synthesised by Dundee Cell Products, based on the HAZV N strain JC280 (Genebank accession number: M86624.1) and was provided in the pET28-SUMO expression vector. The HAZV N ORF was sub-cloned into the expression vector pGEX6P2 using 5’ BamHI and 3’ XhoI restriction sites such that HAZV N was expressed as an N-terminal GST fusion protein (GST-HAZV N). Protein expression was induced in the *E.coli* strain Rosetta 2 using 100 μM isopropyl β-D-1-thiogalactopyranoside (IPTG) at 18 °C for 16 h. GST-HAZV N was then extracted using glutathione affinity chromatography: bacterial cells were pelleted by centrifugation, and bacterial proteins extracted by incubating in lysis buffer (100 mM NaCl, 20 mM Tris pH 8.0, 1 % (v/v) Triton X-100, 1 mg/mL chicken egg white lysozyme (Sigma Aldrich), 1 mM MgCl_2_, 1x complete protease inhibitor cocktail EDTA-free (Roche), 1 unit (U) DNase and 1 U RNase) on ice for 30 min, followed by repeated rounds of sonication. Cell lysates were then clarified by centrifugation at 18,000 x g for 30 min at 4 °C and the supernatant was applied to GST resin that had been pre-equilibrated in binding buffer (100 mM NaCl, 20 mM Tris pH 8.0). GST-HAZV N was allowed to bind to GST resin for 1 h at room temperature, then the unbound fraction was collected and the resin washed once in 4 volumes of binding buffer, once in 4 volumes binding buffer containing 1.5 M NaCl to remove RNA derived from the bacterial expression host, then resin was washed twice more in 4 volumes of binding buffer. Elution buffer (40 mM reduced glutathione, 5 % glycerol, 50 mM NaCl, 15 mM HEPES pH 7.0, 1 mM DTT) was used to dissociate GST-HAZV N from the resin in 3 successive elutions. The GST tag was removed by overnight incubation with HRV 3C protease, then size exclusion chromatography (SEC) was performed to analyse the oligomeric status of HAZV N using a HiLoad 26/600 Superdex 75 pg column (GE Healthcare) with an Akta Prime pump system at 4 °C.

### Ion exchange chromatography

Cation exchange chromatography was performed using a Resource S column at 4 °C with all buffers at pH 7.0. The Resource S column was equilibrated in binding buffer containing 50 mM NaCl, 15 mM HEPES pH 7.0, 1 mM DTT, and the GST-HAZV N, HAZV N and GST protein mixture was applied to the column. Binding buffer was then run through the column until the 280 nm trace returned to baseline. Elution buffer containing 1 M NaCl, 15 mM HEPES pH 7.0, 1 mM DTT was applied to the column in a linear gradient from 0 to 100 % over 25 column volumes, in order to elute bound HAZV N.

### Circular dichroism

Both CCHFV and HAZV N (15 μM monomer concentration) proteins were dialysed into 100 mM NaCl, 20 mM H_2_NaO_4_P pH 7.0 and transferred to 1 mm path-length quartz cuvettes. Thermal melts were carried out in a Chirascan Spectrometer (Applied Photophysics) monitoring ellipticity at 222 nm as the temperature of the samples was increased from 10 to 87 °C in 1 °C intervals at 1 °C/min using 4.3 nm bandwidth. The data were fit to a Boltzmann sigmoid using Graphpad Prism v6.

### Crystallisation, data collection and structure solution

Crystallisation trials were performed using the sitting-drop vapour-diffusion method in MRC plates (Molecular dimension) using HAZV N purified by ion exchange chromatography and concentrated to 10 mg mL^−1^. An Oryx 6 crystallisation robot (Douglas Instruments) was used to determine conditions under which crystals of HAZV N grew using multiple commercially available sparse matrix screens including Index, Crystal Screen, Crystal Screen 2, Salt RX (Hampton Research), Wizard 1 and Wizard 2 (Emerald BioSystems). Each screen was incubated at either 11, 18, or 25 °C with protein:mother liquor ratios of 50:50 and 70:30. Crystals of HAZV N were observed in several wells, however crystals that grew in 15 % (w/v) polyethylene glycol (PEG) 10,000, 100 mM sodium citrate pH 5.5, 2 % (v/v) dioxane looked most promising, and this condition was further optimised. The concentration of PEG 10,000, type of PEG, pH and salts were varied in sequential rounds of optimisation using the hanging-drop vapour-diffusion method, and ultimately crystals grown in the following condition (15 % PEG 10,000, 100 mM sodium citrate pH 5, 100 mM NaCl, 2 % dioxane) provided the diffraction data for HAZV N.

30 % PEG 200 was used as a cryo-protectant for HAZV N crystals, prior to cryo-cooling in liquid nitrogen, and the diffraction data was subsequently recorded on beamline I02 at the Diamond Light Source (UK) at 100 K. The best data recorded diffracted to a resolution of 2.7 Å in the P2_1_2_1_2_1_ space group. X-ray data for HAZV N crystals was indexed and integrated using XDS [31] and scaled using AIMLESS [32]. All data collection and scaling statistics are shown in Table 1. The crystal structure of HAZV N was determined by molecular replacement using the program PHASER [33] with the globular domain of CCHFV N (PDB accession number: 4AKL, [18]); residues 1–182, and 296–482 as the search model. A preliminary structure was auto-built into the electron density maps with BUCANEER [34]. Model building and refinement were carried out using COOT [35] and REFMAC5 [36] and the geometry of the final models checked using MolProbity [37]. The final structure was refined to R_cryst_ = 24.2 % and R_free_ = 28.1 %. A summary of the refinement statistics is presented in Table 1. All figures were generated using PyMol.

**Table 1:** Data collection, scaling and refinement statistics

Dataset	Apo-HAZV N
Wavelength (Å)	(i02) 0.9795
Space group	P2_1_2_1_2_1_
Cell parameters (Å,°)	a = 64.99; b = 76.10; c = 449.30 α = 90; β = 90; γ = 90
Total reflections	252499 (17368)
Unique reflections	62009 (4410)
Resolution shells (Å)	
Low	74.88–2.70
High	2.77–2.70
*R*_*merge*,_ %	11.6 (63.1)
R_pim_ %	8.6 (50.8)
Completeness, %	98.9 (98.2)
Multiplicity	4.1 (3.9)
*I/σ(I)*	9.3 (1.7)
R_cryst_ %	24.18
R_free_ %	28.12
V_M_	2.57
Mol. Per AU	4
Reflections working set	58670
Free R-value set (no. of reflections)	5.1 % (3125)
RMSD bond lengths (Å)	0.013
RMSD bond angles (°)	1.553
No. atoms used in refinement	
Non-hydrogen atoms	15226
Protein atoms	15156
Water molecules	70
RNA atoms	0
Mean B factor, Å^2^	
Total	44.90
Protein atoms	44.7
Water molecules	51.1
Ramachandran plot statistics, %	
Preferred region	96.49
Allowed region	2.88
Outliers	0.27
Molprobity clashscore for all atoms	2.85

Parentheses indicate the corresponding highest resolution shell values

### Availability of supporting data

The atomic coordinates and structure factor amplitudes are available in the Protein Data Bank repository (PDB), Accession Code 5a97.

## Abbreviations

HAZV: Hazara virus
CCHFV: Crimean-Congo haemorrhagic fever virus
N: Nucleocapsid protein
GST: Glutathione S-transferase
SNS: Segmented negative stranded
RNP: Ribonucleoprotein
